# Major challenges of integrating agriculture into climate change mitigation policy frameworks

**DOI:** 10.1007/s11027-017-9743-2

**Published:** 2017-04-12

**Authors:** Thomas Fellmann, Peter Witzke, Franz Weiss, Benjamin Van Doorslaer, Dusan Drabik, Ingo Huck, Guna Salputra, Torbjörn Jansson, Adrian Leip

**Affiliations:** 1 European Commission, Joint Research Centre, Directorate Sustainable Resources, Seville, Spain; 2 EuroCARE, Bonn, Germany; 3 European Commission, Joint Research Centre, Directorate Sustainable Resources, Ispra, Italy; 4 Agricultural Economics and Rural Policy Group, Wageningen University, Wageningen, The Netherlands; 5 Department of Economics, Swedish University of Agricultural Sciences, Uppsala, Sweden

**Keywords:** Agriculture, Climate change, Emissions, Mitigation, Policy

## Abstract

Taking the European Union (EU) as a case study, we simulate the application of non-uniform national mitigation targets to achieve a sectoral reduction in agricultural non-carbon dioxide (CO_2_) greenhouse gas (GHG) emissions. Scenario results show substantial impacts on EU agricultural production, in particular, the livestock sector. Significant increases in imports and decreases in exports result in rather moderate domestic consumption impacts but induce production increases in non-EU countries that are associated with considerable emission leakage effects. The results underline four major challenges for the general integration of agriculture into national and global climate change mitigation policy frameworks and strategies, as they strengthen requests for (1) a targeted but flexible implementation of mitigation obligations at national and global level and (2) the need for a wider consideration of technological mitigation options. The results also indicate that a globally effective reduction in agricultural emissions requires (3) multilateral commitments for agriculture to limit emission leakage and may have to (4) consider options that tackle the reduction in GHG emissions from the consumption side.

## Introduction

In anticipation of the conclusion of the Paris Agreement on Climate Change, countries were asked to submit Intended Nationally Determined Contributions (INDCs) for climate action to the United Nations Framework Convention on Climate Change (UNFCCC). The agreement legally entered into force on 4 November 2016 after the threshold requirements were met. Although specific modalities and procedures still have to be negotiated, the INDCs are set to become Nationally Determined Contributions (NDCs) and will form the basis for implementation for the parties that ratified the Paris Agreement. As of October 2016, 162 INDCs were submitted, representing a total of 189 countries as the European Union (EU) submitted a single INDC for all of its 28 member states. Agriculture is mentioned in 121 countries as one of the sectors where emission reductions are intended, but so far only a few of them set quantitative targets for agriculture (Richards et al. 2016; UNFCCC 2016).

**Fig. 1:**
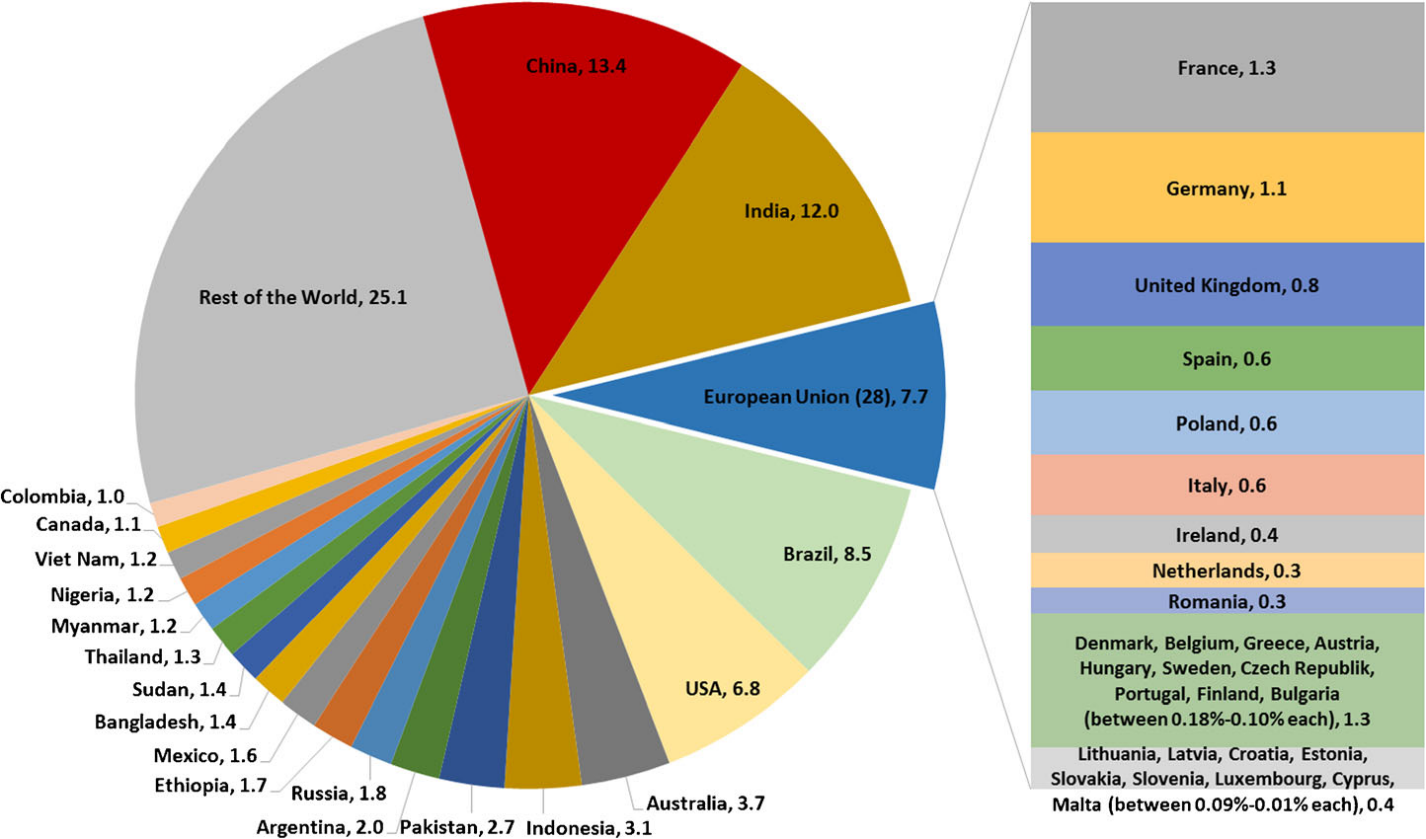
Share in global agriculture GHG emissions (%, 2012). Source: FAO (2016)

The agricultural sector is a large contributor of non-carbon dioxide (CO_2_) greenhouse gas (GHG) emissions, namely methane (CH_4_) and nitrous oxide (N_2_O) from livestock, manure management, fertilizer use, rice (*Oryza*) cultivation, agricultural soils, burning of crop residues and savannahs. Agriculture contributes between 10 and 12% of global GHG emissions (Smith et al. 2014).^1^ Figure 1 shows that China (13.4%), India (12%), Brazil (8.5%), the United States of America (USA) (6.8%) and the aggregated 28 member states of the EU (7.7%) together account for almost 50% of global agriculture emissions. However, from the EU, only France (1.3%) and Germany (1.1%) belong to the 22 countries with a share exceeding 1.0% of global agriculture GHG emissions. On the other hand, with respect to the EU, France (19%), Germany (15%) and the UK (11%) together account for about 45% of total EU agriculture emissions, with the next highest contributions from Spain, Poland and Italy (8% each).

**Fig. 2:**
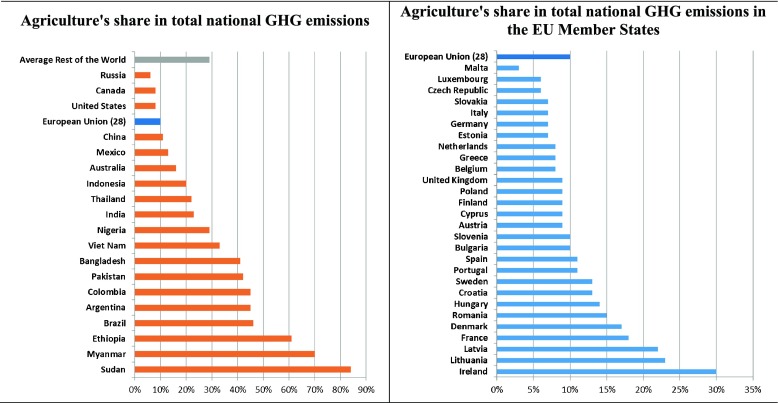
Share of agriculture in total national GHG emissions (%). The *left panel* depicts the countries with a share of at least 1.0% in global agriculture GHG emissions (cf. Fig. 1). Source: Own compilation based on data of Richards et al. (2015)

Depending on the relative size and importance of the agricultural sector, the share of agriculture in total national GHG emissions varies substantially among countries. Analysing data from National Communications to the UNFCCC, Richards et al. (2015) found that on average, agriculture contributes about 30% to national GHG emissions (excluding emissions from land use, land use change and forestry (LULUCF)). This is higher than the estimated 10–12% contribution of agriculture to global emissions because of a large number of countries where agriculture emissions are relatively important in national GHG emissions even though they are rather small in absolute terms. In 42 low-income developing countries, agriculture contributes more than 50% to national emissions, whereas on average it is 35% in developing countries and 12% in developed countries (Richards et al. 2015). Figure 2 shows that in the major contributors to global agriculture emissions (presented in Fig. 1), the share of agriculture in national GHG emissions is quite diverse (e.g. 46% in Brazil, 23% in India, 11% in China and 8% in the USA). In the EU as a whole, agriculture contributes about 10% to total GHG emissions, but respective shares in the member states are also very diverse: the highest in Ireland (31%) and the lowest in Malta (2.5%).

Notwithstanding the diversity of the absolute and relative importance of agriculture emissions, recent scenario analyses indicate that reductions in agricultural emissions will be important for meeting ambitious global climate goals of limiting warming to 1.5 or 2 °C above pre-industrial levels (Reisinger et al. 2013; Gernaat et al. 2015; Wollenberg et al. 2016). Therefore, the Paris Agreement has put the agricultural sector back into focus when it comes to the fine-tuning of how countries achieve their overall emission reduction targets.

Against this background, the purpose of this paper is to highlight and discuss potential impacts and major challenges of integrating agriculture into national and international climate change mitigation policy frameworks and strategies. More precisely, we want to address two questions: What would be the effects on production, prices, income, consumption, trade and emission leakage if countries rigidly applied national GHG mitigation targets onto their agriculture sector? What are the major challenges that have to be tackled for an efficient and effective emission reduction policy in the agriculture sector? To address these questions, we take the EU as case a study. There are at least two reasons for this selection: (i) the EU shows a very diversified structure of the absolute and relative importance of agricultural emissions within its member states, reflecting the respective global diversity indicated above and (ii) in its INDC, the EU committed to reducing GHG emissions by at least 40% by 2030 compared to 1990 levels (European Council 2014). Similar to most other parties to the Paris Agreement, specific EU legislative measures still have to be negotiated, including national mitigation targets for the member states and the specific way the agricultural sector will be included in the mitigation strategy. Therefore, we model an illustrative scenario that simulates a rigid implementation of non-uniform mitigation targets for EU agriculture according to a distribution key that is based on current national targets for the EU member states. Scenario results allow us to highlight and discuss key challenges significant not only for the EU but also relevant globally with respect to the integration of agriculture into national and global climate change mitigation strategies.

## Modelling approach and scenario setting

For the quantitative analysis, we employ an adjusted version of the Common Agricultural Policy Regional Impact Analysis (CAPRI) modelling system. CAPRI is a large-scale economic comparative-static, global multi-commodity, agricultural sector model. The model is frequently used for assessing the impact of agricultural, environmental and trade policies on agricultural production, trade, prices and income as well as environmental indicators in a consistent framework. Although the model focuses on the EU (on regional, member state and aggregated EU level), it is a global model as it covers global bilateral trade for major agricultural commodities. CAPRI consists of two interacting modules: a supply module and a market module. The supply module comprises independent aggregate optimization models representing agricultural activities (28 crop and 13 animal activities) in all 273 Nomenclature of Territorial Units for Statistics (NUTS)2 regions within the EU. The market module consists of a spatial, global multi-commodity model for 47 primary and processed agricultural products, covering 77 countries in 40 trade blocks. The behavioural functions for supply, human consumption, feed and processing in the market module are all differentiated by commodity and geographical units and apply flexible functional forms so that calibration algorithms ensure full compliance with microeconomic theory. The link between the supply and market modules is based on an iterative procedure until an equilibrium is obtained (Britz and Witzke 2014; CAPRI 2016).

The regional supply models in CAPRI capture links between agricultural production activities in detail. Based on the inputs and outputs of these activities, agricultural GHG emissions are endogenously calculated according to the IPCC (2006) tier 2 method for the most important drivers. For activities where the respective information is missing, a tier 1 approach is applied to calculate GHG emissions (e.g. rice cultivation). The CAPRI reporting of agriculture GHG emissions mimics the reporting of emissions by the EU to the UNFCCC, that is, a Global Warming Potential (GWP) of 21 for methane and 310 for nitrous oxide is assumed. A more detailed description of the general calculation of agricultural emission inventories in CAPRI on activity level is given in Pérez Domínguez (2006), Leip et al. (2010) and Pérez Domínguez et al. (2012).

In previous GHG mitigation policy analyses with CAPRI, technological (i.e. technical and management-based) mitigation options were not implemented endogenously. This paper draws on the first study to endogenize the choice among the following selected technological mitigation options (each of them can voluntarily be applied by farmers) within the CAPRI model (see Van Doorslaer et al. 2015): (1) farm scale anaerobic digestion, (2) community anaerobic digestion, (3) nitrification inhibitors, (4) improved timing of fertilization, (5) precision farming and (6) changes in the composition of animals’ feed. The model allows the simultaneous use of different options, for example, nitrification inhibitors, the timing of fertilization and precision farming can be combined to reduce N_2_O emissions due to fertilizer applications. Implementation costs and mitigation potential of the modelled technological mitigation options are taken from the Greenhouse Gas and Air Pollution Interactions and Synergies (GAINS) database (GAINS 2013; Höglund-Isaksson et al. 2013). The level of production activities and the use of mitigation technologies are constrained by various factors, including land availability, fertilization requirements of the cropping systems versus organic nutrient availability, feed requirements in terms of dry matter, net energy, protein and fibre for each animal. Production activities and decision-making are also influenced by agricultural and environmental policy restrictions (Britz and Witzke 2014; CAPRI 2016). The next section provides more details on how mitigation options have been implemented into the CAPRI model.

Emission reductions in the EU may be linked to production decreases that can trigger increases in imports or decreases in exports. This can induce production increases in non-EU countries, leading to higher emissions in these regions (i.e. emission leakage). To estimate emission impacts outside the EU, a specific CAPRI module has been further developed to estimate emission factors for agricultural products for non-EU countries. The module applies fixed emission coefficient to agricultural production outside of the EU, as computed by the market model. The emission coefficients were estimated using time series data on agricultural production from the Food and Agriculture Organization Corporate Statistical Database (FAOSTAT) and emission inventories from the Emission Database for Global Atmospheric Research (EDGAR 2013). A robust estimator was developed within a Bayesian framework, using prior distributions for coefficients obtained from the bottom-up computation of emissions in the CAPRI supply model. Once estimated, the coefficients were fixed in simulations, thus permitting no endogenous response in emission intensity in a simulation. As for the calculation of EU agricultural emissions in this paper, we also consider only emissions of the UNFCCC category ‘Agriculture’ for the approach to quantifying emission leakage, that is, other emissions related to agricultural production, like, for example, emissions from land use change, are not covered (for a detailed description of the CAPRI emission leakage methodology see Jansson et al. 2010, Pérez Domínguez et al. 2012 and Van Doorslaer et al. 2015).

### The model set-up

The regional income maximization is formulated as

1 $$ \begin{array}{l} \max \kern0.5em  R\left(\mathrm{act}\right)-{C}^{\mathrm{T}}\left(\mathrm{act},\mathrm{fert},\mathrm{feed},\mathrm{mshar}\right)\\ {}\mathrm{s}.\mathrm{t}.\\ {} G\left(\mathrm{act},\mathrm{feed},\mathrm{fert}\right)\le 0\\ {}0\le {\mathrm{mshar}}_{a, m, e}\le 1,\forall m\\ {}{\sum}_m{\mathrm{mshar}}_{a, m, e}=1,\end{array} $$

where the regional indices are omitted and

*R* revenue function, combining sales from marketable outputs from production activities as well as premiums directly paid to activities,

*C* ^T^ total cost function, combining cost elements directly related to activities, as well as purchases of marketable inputs (feed, fertilizer) and costs of mitigation efforts,

*G*vector constraint function representing agricultural technology,

*act* vector of production activities with a certain intensity. Typical element: act_*a*_,

*a* set of production activities (e.g. dairy cows with high yield),

*fert* vector of mineral fertilizer purchases. Typical element: fert_*n*_,

*n* set of plant nutrients (N, P, K),

*feed* matrix of feed input coefficients. Typical element: feed_*a,f*_,

*f* set of feed items (e.g. feed cereals),

*mshar* vector of mitigation shares. Typical element mshar_*a,m,e*_,

*m* set of mitigation technologies (including ‘no mitigation’),

*e* set of emission types (e.g. CH_4_ from manure management).

The cost function is assumed to be separable into parts related to mitigation efforts and other costs

2 $$ \begin{array}{c}{C}^{\mathrm{T}}\left(\mathrm{act},\mathrm{fert},\mathrm{feed},\mathrm{mshar}\right)={\sum}_a{\mathrm{act}}_a{\sum}_{m, e}{C}^m\left({\mathrm{mshar}}_{a, m, e}\right)+{\mathrm{fert}}_N{\sum}_m{C}^m\left({\mathrm{mshar}}_{N, m,{\mathrm{N}}_2\mathrm{O} \min}\right),\\ {}+{C}^O\left(\mathrm{act},\mathrm{fert},\mathrm{feed}\right)\end{array} $$

where

*C* ^*m*^ mitigation cost per activity level for mitigation option *m*, which depends on mitigation share *mshar*_*a,m,e*_ for activity *a*, mitigation option *m* and targeting emission type *e*,

*C* ^*O*^other (non-mitigation) cost depending on activity level, feed coefficients and fertilizer quantities.

The mitigation shares do not enter the constraint function G(.) nor the cost function *C* ^O^. In the case of anaerobic digestion (AD), a relevant mitigation technology targeting CH_4_, this seems to be largely correct, assuming the residues (containing the nitrogen and other plant nutrients from the manure and other feedstock for AD) are returned to the soil without significant losses. The only effect of AD is then to reduce CH_4_ emissions from manure and to generate income (negative cost *C* ^*m*^). The assumption of no influence of mitigation on constraints and other costs is more questionable for measures to reduce N_2_O emissions from fertilizer application such as precision farming or improved timing of fertilization. These measures should also influence the overall nutrient balance in the crop sector, but this is currently neglected in our modelling approach.

Most emission types are calculated as the product of emission factors per activity level (determined as a function of yields and other characteristics) and activity levels. For some of them, mitigation measures may reduce emissions according to a factor *mfac*_*a,e*_ below the standard, uncontrolled amount (=100%). The most important example is the reduction in CH_4_ emissions from manure management according to the GAINS (2013) mitigation options farm scale and community scale anaerobic digestion plants. Formally,

3 $$ \begin{array}{l}{\mathrm{emi}}_e={\sum}_a{\mathrm{mfac}}_{a, e}\times {\varepsilon}_{a, e}\times {\mathrm{act}}_a\\ {}\mathrm{where}\kern17em ,\\ {}{\mathrm{mfac}}_{a, e}={\sum}_m\;{\mu}_{a, m, e}\times {\mathrm{mshar}}_{a, m, e}\end{array} $$

and

*emi*_*e*_ emissions of type *e*,

*ε*_a,e_ uncontrolled emission factor for emission type *e* from activity *a*,

*μ*_a,m,e_ reduction factor for emission type *e* from activity *a*, if a certain mitigation technology *m* were fully implemented (which may be infeasible).

Emissions of N_2_O from synthetic fertilizers are incorporated similarly with the total use of mineral fertilizer adopting the role of emissions causing activity. Relevant emission mitigation options are nitrogen inhibitors, timing of fertilization and precision farming, as defined in the GAINS model (the mitigation technologies can also be combined)

4 $$ \begin{array}{l}{\mathrm{emi}}_{{\mathrm{N}}_2\mathrm{O} \min }={ m fac}_{N,{\mathrm{N}}_2\mathrm{O} \min}\times {\varepsilon}_{N,{\mathrm{N}}_2\mathrm{O} \min e}\times {\mathrm{fert}}_N\\ {}\mathrm{where}\kern29em .\\ {}{\mathrm{mfac}}_{N,{\mathrm{N}}_2\mathrm{O} \min }={\sum}_m\;{\mu}_{N, m,{\mathrm{N}}_2\mathrm{O} \min}\times {\mathrm{mshar}}_{N, m,{\mathrm{N}}_2\mathrm{O} \min}\end{array} $$

Emissions from enteric fermentation per animal category are calculated according to IPCC tier 2 methods from animal numbers, feed intake in gross energy and methane conversion factor. In the CAPRI model, unlike the situation in inventory calculations envisaged by IPCC (2006), feed intake and its composition are known model variables. Therefore, it is possible to directly compute gross energy intake from the endogenous feed input coefficients and thereby capture the effects of endogenous changes in the feed mix on digestibility and emissions. Mitigation factors are applied as above, reflecting the saving of methane emissions if anaerobic digestion plants are used:

5 $$ \begin{array}{l}{\mathrm{emi}}_{{\mathrm{CH}}_4 en}={\sum}_a{\mathrm{mfac}}_{a,{\mathrm{CH}}_4 en}\times {\mathrm{act}}_a\times {\sum}_f{\varepsilon}_{a, f,{\mathrm{CH}}_4 en}\times {\mathrm{feed}}_{a, f}\\ {}\mathrm{where}\kern32.5em .\\ {}{\mathrm{mfac}}_{a,{\mathrm{CH}}_4 en}={\sum}_m\;{\mu}_{a, m,{\mathrm{CH}}_4 en}\times {\mathrm{mshar}}_{a, m,{\mathrm{CH}}_4 en}\end{array} $$

In summary, the objective of a CAPRI supply model is to maximize the net revenues as in Eq. (1), considering given parameters such as product prices and premiums paid under the EU’s Common Agricultural Policy, as well as the costs for technological mitigation measures and other costs. Following an iterative procedure, the model obtains an equilibrium that reflects the optimum of production activities, mitigation technologies and feed use for a given emission target.

### Construction of the scenarios

We construct a reference scenario (REF) and a mitigation policy scenario (HET28) to simulate a rigid implementation of mitigation targets for the EU agricultural sector.^2^ The simulation year for both scenarios is 2030, and in both scenarios, farmers can voluntarily apply the above-mentioned technological GHG mitigation options. The REF scenario assumes the status quo policy as scheduled in the current legislation based on the information available by mid-2015 (e.g. abolishing the milk and sugar quotas). The REF scenario relies on the agricultural market outlook of the European Commission (2012), which itself is based on the Agricultural Outlook of the Organization for Economic Cooperation and Development (OECD) and the Food and Agriculture Organization of the United Nations (FAO) (OECD-FAO 2012) and gives medium-term projections up to the year 2022 in a consistent analysis framework by using also external sources for the assumptions on macroeconomic developments (like GDP growth, exchange rates, world oil prices and population growth). As the projection year for our analysis is 2030, we extrapolated and supplemented the European Commission’s projections with other information to arrive at the CAPRI REF results for the year 2030 (for more information on the CAPRI baseline process, see Himics et al. 2014).

With respect to GHG emission mitigation obligations, the EU agricultural sector is included under the Effort Sharing Decision (ESD) within the ‘2020 Climate and Energy Package’ of the EU (European Council 2009). In this ESD, the EU member states have non-uniform GHG emission mitigation targets based on the relative gross domestic product (GDP) per capita. However, the mitigation targets are specific to the member states, but not individual sectors, and up to now, no explicit policy measures have been implemented to force GHG emission abatement in the agriculture sector. Therefore, no mitigation targets are applied in the REF scenario.

**Fig. 3:**
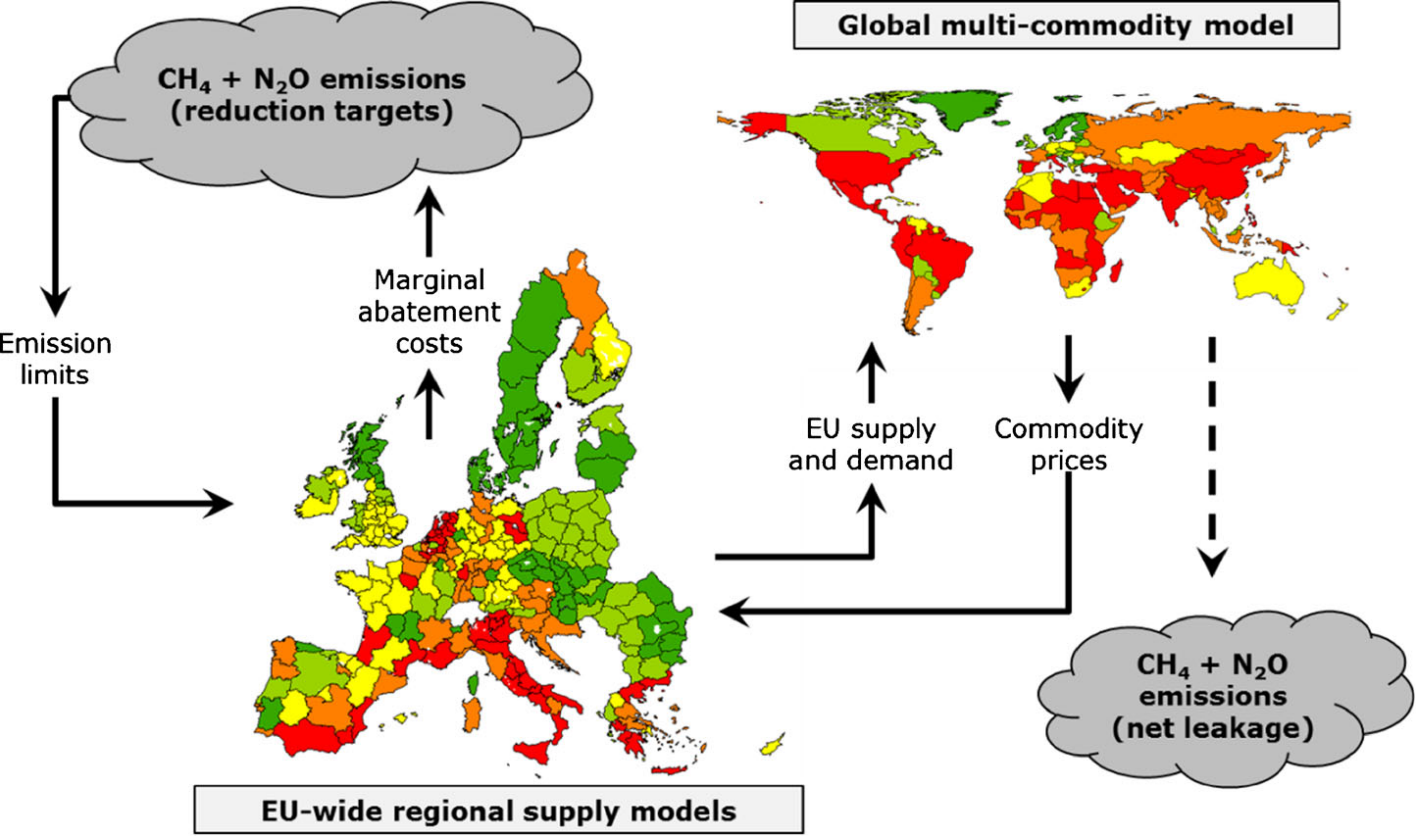
CAPRI model structure and implementation of the mitigation policy scenario. Source: Adjusted from Pérez Domínguez and Fellmann (2015)

The mitigation policy scenario (HET28) follows the general setting of the REF scenario but aims at an EU-wide reduction in agricultural non-CO_2_ emissions of 28% in the year 2030 compared to 2005. The 28% reduction target is in line with the European Commission’s roadmap for moving to a low-carbon economy in 2050 and an accompanying impact assessment (European Commission 2014a, b). It has to be noted that in the new ‘2030 Climate and Energy Framework’ of the EU, submitted as INDC to the UNFCCC, agriculture emissions are again covered in an ESD, but targets for the member states for 2030 are still under discussion (European Council 2014). For our HET28 scenario, we, therefore, use mitigation targets for 2030 that are based on national targets of the current EU ESD (European Council 2009). The current ESD aims at a total EU GHG reduction of 20% by 2020 compared to 2005 emission levels. However, the ESD covers several sectors and applying the specific member states targets just to agriculture would translate to a total reduction in EU agriculture emissions by only 9%. Therefore, we increase the member states mitigation targets according to a linear modification (ESD −19%), such that a 28% reduction in agricultural non-CO_2_ emissions is achieved at the aggregated EU level (Table 1). The corresponding emission reduction obligations are set per EU member state and NUTS2 region. The CAPRI model structure and the implementation of the mitigation scenario are depicted in Fig. 3.

**Table 1:** GHG emission reduction target for agriculture in the EU member states in 2030 compared to 2005, as assumed in the HET28 scenario

Member state(EU-15)^a^	Agricultural emission target	Member state(EU-N12)^b^	Agricultural emission target
Austria	−35	Bulgaria	+1
Belgium-Lux.	−34	Cyprus	−24
Denmark	−39	Czech Republic	−10
Finland	−35	Estonia	−8
France	−33	Hungary	−9
Germany	−33	Latvia	−2
Greece	−23	Lithuania	−4
Ireland	−39	Malta	−14
Italy	−32	Poland	−5
Netherlands	−35	Romania	0
Portugal	−18	Slovak Republic	−6
Spain	−29	Slovenia	−15
Sweden	−36		
UK	-35	EU	−28

The member state targets are based on the current EU Effort Sharing Decision (European Council 2009), increased according to a linear modification (ESD −19%) such that a 28% reduction in total EU agricultural non-CO_2_ emissions is achieved

^a^EU-15: 15 EU member states before 2004

^b^EU-N12: 12 EU member states of the 2004 and 2007 enlargements (Croatia has not been included in the analysis)

## Scenario results

**Fig. 4:**
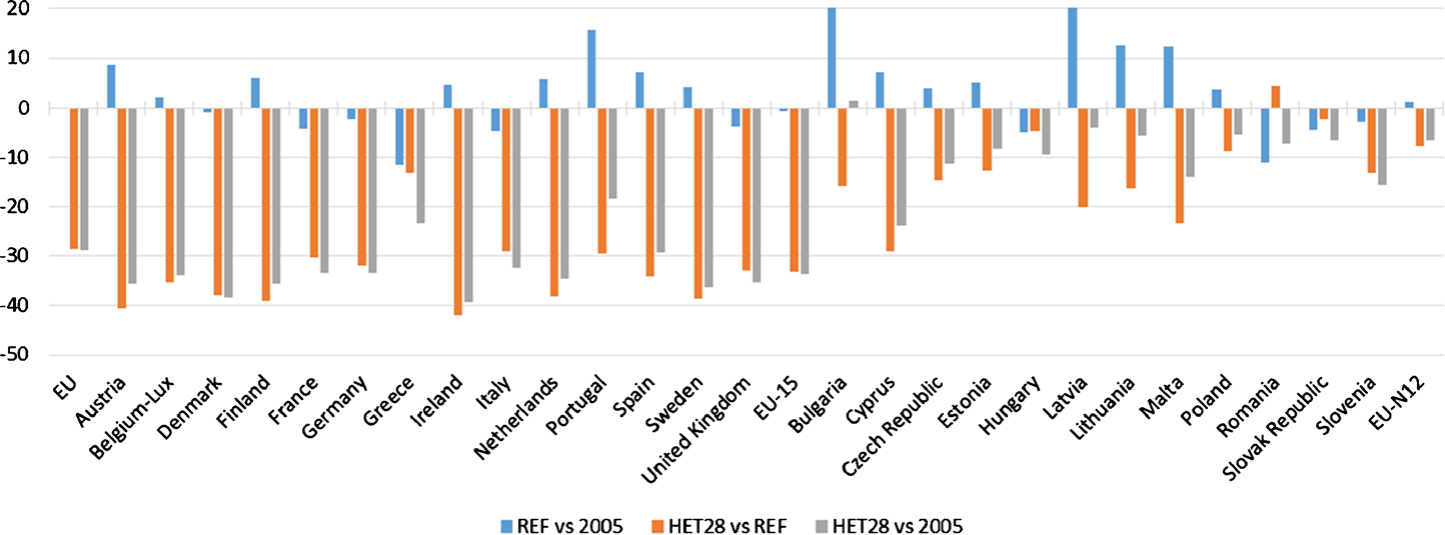
Percentage changes in agriculture GHG emissions per EU member state (2030). The year 2005 is an ex-post simulation close to the historical 2005 data. EU-15: 15 EU member states before 2004 and EU-N12: 12 EU member states of the 2004 and 2007 enlargements

Figure 4 presents the decomposition of the EU agriculture GHG emission developments under the REF and HET28 scenarios. The REF scenario shows agriculture’s GHG emissions with no specific emission reduction requirements in place, indicating that by 2030 agriculture emissions in the EU are almost equal to the levels of 2005 (+0.2%). This is especially notable when considering that between 1990 and 2012 the sector experienced a rather steady downward trend of −24% (compared to −19% for total EU emissions, excluding LULUCF). This historical decrease is mainly attributable to reductions in livestock numbers and productivity increases, as well as the implementation of agricultural and environmental policies (Van Doorslaer et al. 2015). The REF scenario projection suggests that agriculture emissions would plateau in 2030, which is a result of the general policy and technology developments and a favourable agricultural market environment. However, results are quite diverse among the member states. Agriculture emissions decrease most in Greece (−12%), Romania (−11%), Italy and Hungary (−5% each), whereas eight member states show increasing emissions, with highest increases indicated for Bulgaria and Latvia (both about +20%) and Portugal (+16%).

The emission reductions at member state level in the HET28 scenario should be seen in the context of the emissions in the REF scenario and the emission reduction obligation a member state faces according to the modelled policy (as indicated in Table 1). By scenario design, the 28% reduction target for EU agriculture emissions is met, and also each member state (at least) meets its respective national mitigation targets. In the following sections, we outline how the EU mitigation obligations affect agricultural production, prices, income, trade, consumption and emissions leakage.

### Impact on EU agricultural production, prices and income

**Fig. 5:**
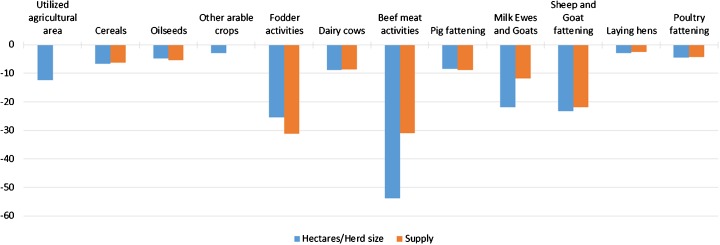
Percentage change in EU area, herd size and supply in the HET28 scenario compared to REF (2030). Supply is not applicable for the aggregates of utilized agricultural area and other arable crops

Figure 5 presents how agricultural activities in the EU are affected in scenario HET28 compared to REF. Most of the adjustments to the GHG mitigation obligation are made through lower activity levels (i.e. decreases in animal numbers, hectares and related supply), with largest reductions projected for the livestock sector, particularly beef meat production. Decreases in area and animal numbers are generally bigger than the decreases in supply, indicating productivity increases per animal and area (least productive areas and livestock are likely to be taken out of production first). Except set aside and fallow land, which is projected to increase by more than 17%, the agricultural area is decreasing for all crops, leading to a total decrease in utilized agricultural area of 12%. In the arable sector, fodder activities decrease the most, which is directly related to the decreases in the livestock sector, in particular to the reduction in the ruminant [namely beef cattle (*Bos taurus*), sheep (*Ovis aries*) and goats (*Capra hircus*)] fattening activities. On the aggregated EU level, the number of beef cattle decreases by almost 54%, and the impacts are most pronounced in those member states that are confronted with the highest mitigation obligations, such as Denmark (82% reduction in beef herd size) and the Netherlands (76%). The impact on dairy cows (*Bos taurus*) and production is less pronounced than on beef cattle and sheep and goat related activities, which can be attributed to a higher profitability of dairy cow production. Furthermore, the impact on pig (*Sus domesticus*) and poultry [namely chicken (*Gallus gallus*), turkey (*Meleagris gallopavo*), duck (*Anas platyrhynchos*) and goose (*Anser anser domesticus*)] fattening is also lower than on ruminant meet production, which is due to their lower emission intensities (i.e. lower GHG emissions per kilogramme meat).

**Fig. 6:**
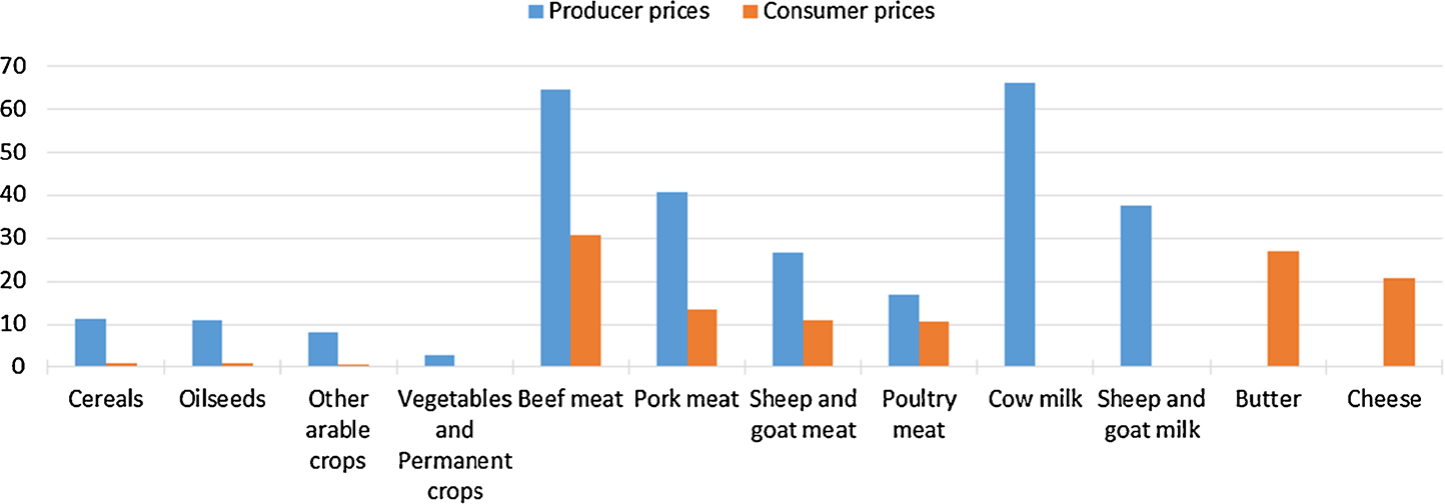
Percentage change in EU producer and consumer prices in the HET28 scenario compared to REF (2030). The consumer price is not applicable for the milk aggregates, whereas the producer price is not applicable for dairy products (butter, cheese)

The general production decrease results in higher producer and consumer prices in the EU in the scenario HET28 compared to REF (Fig. 6). The rise in producer prices is in line with the projected production decreases, showing highest price increases for beef meat (+64%) and cow milk (66%), and increases of about 11% for producer prices of cereals and oilseeds. Consumer price changes are of the same magnitude when looking at absolute changes, but due to high consumer margins (assumed constant), the relative changes are much lower. The relative increases in consumer prices for meat and dairy products vary between 10 and 30%, whereas the impact on consumer prices for crops is below 1%.

The production and price developments affect agricultural income. Total agricultural income takes into account the changes in the product margins (gross value added less cost) and in the production quantity of all agricultural activities. The scenario results indicate that in about 95% of the EU regions the increase in producer prices more than offset the income losses provoked by production decreases and increases in production costs. As a consequence, total agricultural income in the EU is projected to increase. However, the aggregated EU result hides large differences between the regions in the member states. Moreover, it has to be kept in mind that the CAPRI model applied is a market model, not a farm model, and therefore cannot depict structural change regarding the number of farmers. Given the large decreases in hectares and herd sizes, it is likely that some (especially smaller and less competitive) farmers would have to leave the sector if they are not able to cope with the rigid mitigation obligation implemented in the scenario. Evidently, only farmers remaining in the sector would benefit from potential income increases.

### Impact on the EU trade balance, consumption and emission leakage

**Fig. 7:**
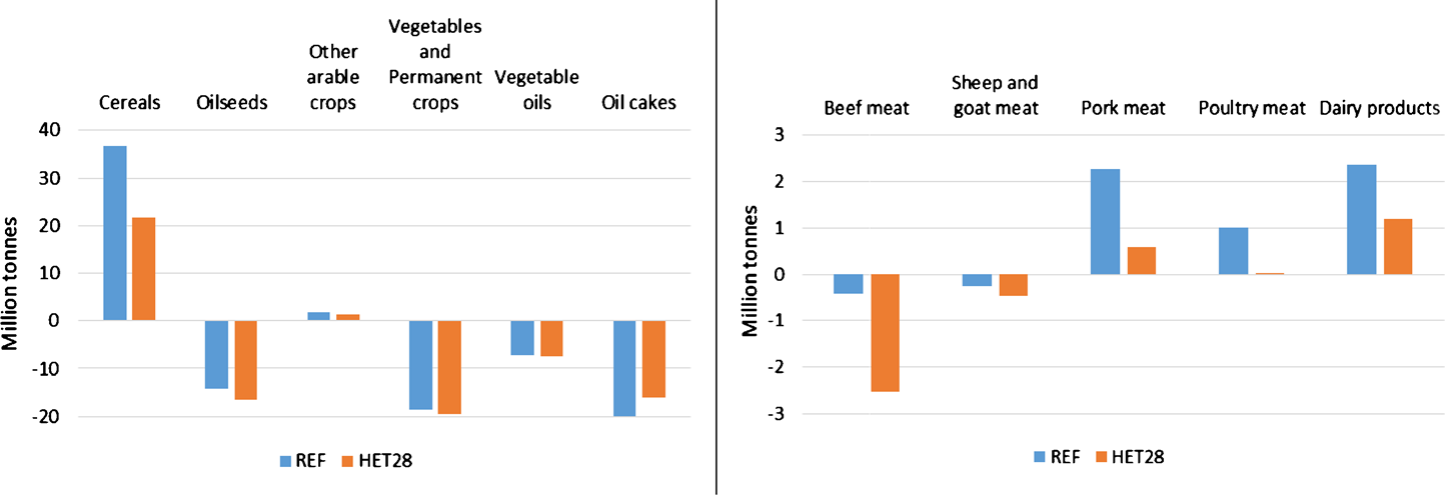
EU trade balance in the REF and HET28 scenarios (2030). Trade balance = exports − imports

The changes in EU production and prices lead to changes in the EU trade balance (exports–imports) as presented in Fig. 7. Regarding the EU’s trade balance, it has to be noted that the scenarios are run under the assumption that current trade agreements and EU border protection mechanisms would stay in place by 2030. Following the large production drops in the EU, almost all agricultural EU exports decrease while at the same time imports increase, leading to a worsening of the EU trade balance of almost all agricultural products. As an exception, net imports are declining for oil cakes [the solids remaining after pressing oilseeds, namely soybean (*Glycine max*), rapeseed (*Brassica napus*) and sunflower seed (*Helianthus annuus*)], which is due to lower feed demand from the EU livestock sector. In line with the production developments, changes in EU imports and exports are more pronounced in the livestock than in the crop sector. EU beef meat imports are projected to increase by about 2 million tonnes (almost 360%). EU exports of pork (−73%) and poultry meat (−44%) decrease significantly, whereas the respective imports increase (although imports involve relatively small quantities). Moreover, the EU trade balance for dairy products weakens considerably, as especially the EU exports are significantly lower (−31%) compared to the REF scenario.

**Fig. 8:**
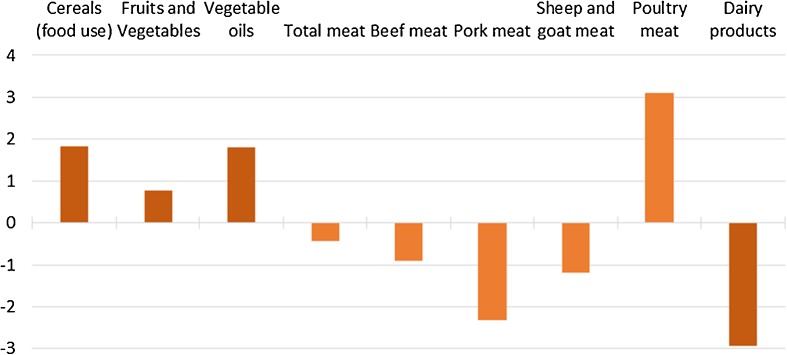
Percentage change in EU consumption in the HET28 scenario compared to REF (2030)

As the increases in imports and the decreases in exports at least partly compensate for the reductions in EU production, the final impact of the GHG mitigation obligation on EU consumption appears to be of relatively lower magnitude (Fig. 8). The biggest consumption decrease is projected for dairy products (almost −3%), varying between −2.5% for fresh milk products and −7.5% for butter (not shown in the figure). Pork meat consumption is expected to decrease by more than 2%, and beef meat as well as sheep and goat meat consumption by about 1%. By contrast, consumers switch to poultry meat, which is less expensive and shows a consumption increase of more than 3%. As a result, total EU meat consumption decreases by only 0.4%. The decreases in the consumption of meat and dairy products seem to be compensated by increases in the food use of cereals (+1.8%), and consumption increases of vegetable oils (+1.8%), and fruits and vegetables (+0.8%).

The changes in the EU trade balance in the HET28 scenario trigger production increases outside the EU, which in turn leads to an increase in agriculture emissions in non-EU countries and hence emission leakage. The effects of the HET28 scenario on global GHG emissions reveal that emission leakage may considerably downsize the net effect of EU mitigation targets on global GHG reduction. Given the model assumptions, the share of EU mitigated emissions offset by emission leakage may be as high as 91%. The major part of emission leakage is caused by EU imports of animal products, with beef and other animal products being responsible for more than 90% of additional emissions outside the EU. According to CAPRI projections, the major part of emission increases outside the EU may happen in Africa, Asia and South America. This is because the EU grants free market access to developing countries in Africa and Asia and because the EU has established trade relations with South America.

## Discussion and conclusions

The Paris Agreement on Climate Change puts the agricultural sector back into focus when it comes to the fine-tuning of how countries achieve their overall GHG emission reduction targets and the way agriculture will be included in national mitigation strategies. We take the EU as a case study and employ a revised version of the CAPRI model to run a reference scenario and an illustrative policy scenario for emission mitigation in agriculture. For the policy scenario, we simulate a rigid implementation of non-uniform mitigation targets for EU agriculture according to a distribution key that is based on augmented current (European Council 2009) national targets for the EU member states. The mitigation target is an EU-wide reduction in agricultural GHG emissions of 28% in the year 2030 compared to the year 2005.

Results of the hypothetical policy scenario show important impacts on EU agricultural production, especially in the livestock sector. Compared to the reference scenario, results of the policy scenario show decreases in the EU cattle numbers of 54%, and in crop and grassland area of up to 12% of the total utilized agricultural area. Crop production is directly affected by the GHG emission reduction obligations and indirectly by the reduced demand for feed from the livestock sector. The decreases in production levels lead to increases in EU agricultural producer prices that are projected to compensate for losses provoked by production decreases and increases in production costs, leading to an increase in total agricultural income at EU level. However, income changes show large regional differences and also some negative income impacts at regional level. Moreover, the model used is a market model and therefore does not account for structural change regarding the number of farms. It is likely that in the policy scenario some (especially smaller and less competitive) farmers would have to leave the sector if they are not able to cope with the GHG mitigation obligations; apparently, only farmers remaining in the sector would benefit from potential increases in total agricultural income. The drop in EU production causes substantially decreasing EU exports and increasing imports, which on the one hand leads to a deterioration of the EU trade balance, but on the other hand, almost compensates for the EU production decreases. Therefore, EU consumption of agricultural products is only moderately affected; for example, total EU meat consumption decreases by only 0.4%, with a slight shift from beef and pork meat consumption to the cheaper and less emission-intensive poultry meat. However, the changes in the EU trade balance induce increases in production and associated emissions in non-EU countries (emission leakage), which considerably downsizes the net effect of the EU mitigation effort.

Our mitigation scenario focuses on the EU, but, as outlined in the introduction, the diversified structure of relative and absolute importance of agricultural emissions within the EU and its member states reflects the respective diversity at the global level. Therefore, our scenario results identify four major challenges that are relevant not only to the EU but generally to all countries with respect to the integration of agriculture into national and global climate policy frameworks and mitigation strategies.

*Challenge 1: Targeted but flexible implementation of mitigation obligations.* Scenario results indicate that it would not be a good strategy if current national mitigation targets were taken as a benchmark to achieve a sectoral EU-wide reduction in agricultural emissions, as this could lead to adverse impacts on agricultural production in most member states and the EU a whole. Our modelling results can be interpreted as high-end estimates of the impacts of the modelled mitigation target for EU agriculture, as farmers are obliged to reduce emissions at the national and regional level, and no grade of flexibility is given regarding the mitigation targets per region. The scenario results suggest that a specific mitigation target for EU agricultural emissions might require a more flexible implementation, also taking into account where emissions are least costly to reduce. Such an approach would not lead to emission reductions according to national mitigation targets nor would it necessarily mean that most emissions would be reduced in those member states with highest absolute agriculture emissions. However, it could help to decrease adverse production effects at aggregated EU level while meeting a specific reduction target for EU agriculture emissions. In general, there is a wide heterogeneity in both mitigation potential and marginal abatement costs within the agricultural sector, not only in the EU but globally, differing between regions and emission sources, and often related to differences in production types and farm size (Vermont and De Cara 2010; MacLeod et al. 2015; Pérez Domínguez and Fellmann 2015; Henderson et al. 2017). A more flexible implementation of mitigation efforts, using market-based approaches, for example, allowing the trade of mitigation obligations among farmers and regions, are considered to diminish adverse effects on production levels (De Cara and Jayet 2011; Pérez Domínguez et al. 2012). Our scenario results suggest that such approaches should be thoroughly assessed before countries implement their mitigation strategies for the agriculture sector.

*Challenge 2: Enhancing the application of technological mitigation options.* For this analysis, a limited set of specific technological (i.e. technical and management-based) GHG mitigation options has been introduced into the modelling approach. In our mitigation scenario, almost all EU crop production would potentially use the provided mitigation options in 2030. On the other hand, based on the included set of options, the impact of a change in livestock production management and technology on overall EU agriculture emissions tends to be rather limited. As a consequence, the largest part of the required GHG reduction is realized by a quantitative adjustment of production (herd size, yield and cultivated hectares). A similar result is likely at global level if the mitigation targets were implemented worldwide. It has to be noted that the modelled set of technologies does not include all available technical and management-based mitigation options (e.g. feed additives to reduce methane emissions from enteric fermentation or genetic improvements steered to increasing milk yields of dairy cows are not modelled). Taking more options into consideration and assuming a wider applicability, say due to additional farm structure change or accelerated technological maturation, could potentially downscale any negative impacts on the EU’s agricultural production and trade in our scenario (see, e.g. Witzke et al. 2014). The importance of technical and management-based options for the mitigation of agricultural emissions is also indicated in the international literature and seems to have particularly great potential for emissions reduction in developing countries (Henderson et al. 2017; Hristov et al. 2013; Smith et al. 2007, 2014). However, it might need specific incentives and support measures (e.g. investment support or training) to trigger and facilitate the implementation of mitigation technologies both within and outside the EU. Moreover, regarding the latter technology transfer may play an important role for emission mitigation, specifically for developing countries (Tilman et al. 2011; Somanathan et al. 2014; Stavins et al. 2014).

*Challenge 3: Reducing emission leakage.* Even though the EU meets its emission reduction target in our policy scenario, an estimate on emission impacts outside the EU shows that the projected EU production decreases go along with emission leakage (i.e. an increase of emissions in non-EU countries), which substantially decreases the global net effect of the EU emissions reduction. The rise in non-EU emissions is due to agricultural production increases triggered to compensate for increasing EU imports and decreasing EU exports. The extent of emission leakage and hence the net gain of national mitigation efforts for global GHG emission reduction depends significantly on the relative GHG efficiency (i.e. emissions per unit of output) of agriculture in the exporting countries compared to the importing country (Caro et al. 2014; Pérez Domínguez and Fellmann 2015; Scott and Barrett 2015). Theoretically, border adjustment measures, like tariffs on imports based on the amount of GHG emissions released in their production, could be a possibility to decrease emission leakage. However, the practical usefulness of such measures is often questioned with respect to their general appropriateness, compliance with rules of the World Trade Organization, and due to concerns about negative welfare effects especially for developing countries (Frankel 2008; Stavins et al. 2014). In any case, such measures might not be necessary if challenges 1 and 2 were successfully tackled, as this would decrease production displacement and hence emission leakage. Moreover, the extent of emission leakage apparently also depends on the commitments other countries make regarding their contributions to the Paris Agreement. It remains to be seen how the global climate agreement will be put into action, but so far, none of the (other) major agricultural trade and important non-CO_2_ emitting countries, like China, Brazil, USA, Australia and Russia, has submitted concrete commitments for the integration of its agricultural sector into binding emission targets (UNFCCC 2016). Our scenario results show that such (multilateral) commitments would not only be necessary in the light of emission leakage and global emission mitigation but also with respect to minimizing distortions to agricultural competitiveness arising from unilateral emission mitigation obligations.

*Challenge 4: Tackling GHG emissions from the consumption side.* Our simulation scenario focuses on agricultural emission reduction from the production side. Scenario results show that the mitigation target set within the EU and related production decreases would affect EU consumers via higher food prices, resulting mainly in a decrease in dairy consumption and a rather slight shift in meat consumption towards poultry meat. However, the consumer price effect is rather low, and human consumption is eventually not significantly affected as the decreased production in the EU is partially compensated for by agricultural and food imports, which in turn jeopardize the mitigation efforts in the EU due to emission leakage. This underlines that it might be necessary to take net imported emissions into account when setting national mitigation targets, which would generally introduce new opportunities for emission reduction strategies on a large scale (Chicco and Stephenson 2012; Scott and Barrett 2015). However, emission reduction targets under the UNFCCC are territory and producer-based, and it is rather unlikely that this approach will be changed shortly (Somanathan et al. 2014). Successfully addressing challenges 1 to 3 would decrease the need for major adjustments regarding consumption patterns. Nevertheless, our scenario results give a strong indication that an effective mitigation strategy to decrease GHG emissions from agriculture should also consider options that tackle the reduction from the consumption side, especially with regard to meat products. GHG emission levels are indeed significantly affected by diets, as protein sources from animals are generally related to higher emissions than vegetable protein sources (Davis et al. 2010), and the importance of a change in consumption patterns to meet stringent climate change targets is also emphasized in the literature (Stehfest et al. 2009; Garnett 2011; Berners-Lee et al. 2012; Hedenus et al. 2014; Stehfest 2014; Westhoek et al. 2014).

Our scenario analysis does not imply that agriculture should be let off the hook with regard to the mitigation of GHG emissions. However, the identified four major challenges need to be tackled to achieve an efficient and effective integration of agriculture into national and international climate change mitigation policy frameworks and strategies.

## Footnotes

1 According to the Common Reporting Format of the UNFCCC, the source category ‘agriculture’ covers the emissions of nitrous oxide and methane. Emissions (and removals) of carbon dioxide (CO_2_) from land use, land use change and forestry (LULUCF) as well as CO_2_ emissions related to energy consumption at farm level (e.g. in buildings and machinery use) or to the processing of inputs (e.g. mineral fertilizers) are attributed to other sectors. In this paper, we focus only on the non-CO_2_ emissions from agriculture.

2 This paper draws on one of the scenarios of the ‘Economic assessment of GHG mitigation policy options for EU agriculture’ (EcAMPA 1) project. For more information on the project and further scenarios, see Van Doorslaer et al. (2015).
